# Can DNA-Based Ecosystem Assessments Quantify Species Abundance? Testing Primer Bias and Biomass—Sequence Relationships with an Innovative Metabarcoding Protocol

**DOI:** 10.1371/journal.pone.0130324

**Published:** 2015-07-08

**Authors:** Vasco Elbrecht, Florian Leese

**Affiliations:** Department of Animal Ecology, Evolution and Biodiversity, Ruhr University Bochum, Universitaetsstrasse 150, D-44801 Bochum, Germany; University of Guelph, CANADA

## Abstract

Metabarcoding is an emerging genetic tool to rapidly assess biodiversity in ecosystems. It involves high-throughput sequencing of a standard gene from an environmental sample and comparison to a reference database. However, no consensus has emerged regarding laboratory pipelines to screen species diversity and infer species abundances from environmental samples. In particular, the effect of primer bias and the detection limit for specimens with a low biomass has not been systematically examined, when processing samples in bulk. We developed and tested a DNA metabarcoding protocol that utilises the standard cytochrome c oxidase subunit I (COI) barcoding fragment to detect freshwater macroinvertebrate taxa. DNA was extracted in bulk, amplified in a single PCR step, and purified, and the libraries were directly sequenced in two independent MiSeq runs (300-bp paired-end reads). Specifically, we assessed the influence of specimen biomass on sequence read abundance by sequencing 31 specimens of a stonefly species with known haplotypes spanning three orders of magnitude in biomass (experiment I). Then, we tested the recovery of 52 different freshwater invertebrate taxa of similar biomass using the same standard barcoding primers (experiment II). Each experiment was replicated ten times to maximise statistical power. The results of both experiments were consistent across replicates. We found a distinct positive correlation between species biomass and resulting numbers of MiSeq reads. Furthermore, we reliably recovered 83% of the 52 taxa used to test primer bias. However, sequence abundance varied by four orders of magnitudes between taxa despite the use of similar amounts of biomass. Our metabarcoding approach yielded reliable results for high-throughput assessments. However, the results indicated that primer efficiency is highly species-specific, which would prevent straightforward assessments of species abundance and biomass in a sample. Thus, PCR-based metabarcoding assessments of biodiversity should rely on presence-absence metrics.

## Introduction

A minor proportion of all species on Earth are known [1]. At the same time, anthropogenic impacts have initiated a mass extinction of species in the “Anthropocene” [2], with pervasive and often negative consequences for ecosystem functioning and human well-being [3,4]. To counteract biodiversity loss, fast and reliable tools are needed to assess and monitor biodiversity [5].

Stream biodiversity is particularity affected by anthropogenic degradation [6,7]. Therefore, large-scale monitoring and management programs have been established, for example, the European Union Water Framework Directive and the US Clean Water Act. In these biomonitoring programs, species lists, particularly of benthic invertebrate indicator species, are the central metric to assess the ecological status of freshwater ecosystems. For stream assessments, hundreds of benthic organisms are sampled in a standardised fashion, sorted, identified, and used in standardised analytical work flows (e.g. [8,9]). However, many benthic invertebrate larvae are difficult to identify at the species level, and thus the most practical taxonomic level for the identification of these organisms is often only the genus or family [10]. This is a major concern, as different species within a genus or subfamily can have different ecological preferences and stress tolerances and belong to different functional feeding groups [11,12] see [13] for review. Even worse, frequent identification errors occur and many specimens are not detected in samples [10]; these limitations have direct consequences for the inferred ecosystem assessment metrics [10,14] and thus management decisions.

DNA barcoding allows for standardized and accurate species identification [15–18]. As this method is DNA based, it can be used to identify species reliably even when juvenile instars or fragments of organisms are available. For animals, a 658-bp standardized fragment of the mitochondrial gene COI (cytochrome c oxidase subunit 1) is typically used [19]. DNA barcoding requires the establishment of an accurate reference database. For macroinvertebrates, this is best achieved by determining diagnostic characters (usually in male adult specimens [13,20,21]), sequencing the specimens, and depositing the COI sequences in a database such as the BOLD database [22]. In times of declining taxonomic expertise [23,24], these curated and public barcode databases are indispensible to conserve taxonomic knowledge.

COI barcoding methods are well established for freshwater organisms [16,17,25] and initial studies have tested their potential for freshwater ecosystem assessments using classical Sanger-based sequencing [14,26]. Stein and co-authors showed that ten of 16 assessment metrics had higher statistical power using DNA barcoding than morphological assessment [14]. However, Sanger sequencing requires that each specimen is processed individually in the laboratory, which is costly and extremely time-consuming for routine community assessments involving hundreds or thousands of specimens per sample.

This challenge can be overcome with the aid of next-generation sequencing, which enables the simultaneous analysis of millions of sequences. One next-generation sequencing technique termed *metabarcoding* (also called *community barcoding*) utilises the same principle as classical barcoding, yet with much higher throughput, allowing the simultaneous processing of hundreds of samples in a single analysis. When complete specimens are identified in bulk, it was suggested to use the term *DNA metabarcoding* to make a distinction to approaches using environmental DNA (eDNA) [27]. However, as our findings largely apply to eDNA-based methods as well, we here refer to metabarcoding in a broad sense. Metabarcoding is currently being tested to address a wide range of biological problems, such as invasive species detection [28], gut content analysis [29], and assessment of microbial [30] and metazoan diversity, such as that of arthropods (e.g. [31,32]). Initial studies on benthic diatoms [33] and macroinvertebrates [34] show the potential of this method to revolutionise the way we monitor stream ecosystems. However, there are general challenges associated with the use of metabarcoding for ecosystem assessments. While preliminary bioinformatic pipelines for data analysis are available (e.g., Mothur [35], QIIME [36], UPARSE pipeline [37]), barcode reference databases are still incomplete. There are furthermore two problems of central importance that have not been addressed systematically. First, sampled organisms have vastly different biomasses, and thus small organisms may be lost owing to low number of sequence reads [38]. Second, the amplification efficiency of the COI gene varies among species, and this might severely bias results [34,39] particularly in view of the variation in biomass. Precise estimates of biomass with respect to specimen recovery and primer bias have not been performed.

We describe an innovative and efficient strategy to analyse macroinvertebrate samples on an Illumina MiSeq sequencing platform. High sequence similarity in in amplicon sequencing can lead to decreased sequence quality on Illumina platforms [40]. We deal with this issue by using uniquely tagged fusion primers targeting the standard barcoding region, which are simultaneously sequenced in forward and reverse sequencing direction to increase nucleotide diversity and thus improve read quality. With the new protocol, we performed two controlled experiments to address the two problems outlined above. First, we assessed the relationship between biomass and sequence abundance by sequencing genetically distinct specimens that differ widely in biomass, but belong to a single species. This allowed us to determine whether and when small specimens are lost owing to low read coverage. Second, we used equal amounts of tissue from 52 freshwater taxa to determine how well they are recovered given species-specific PCR amplification bias when extracting many species in bulk. All analyses were performed with ten replicates to improve statistical robustness.

## Materials and Methods

**Fig 1:**
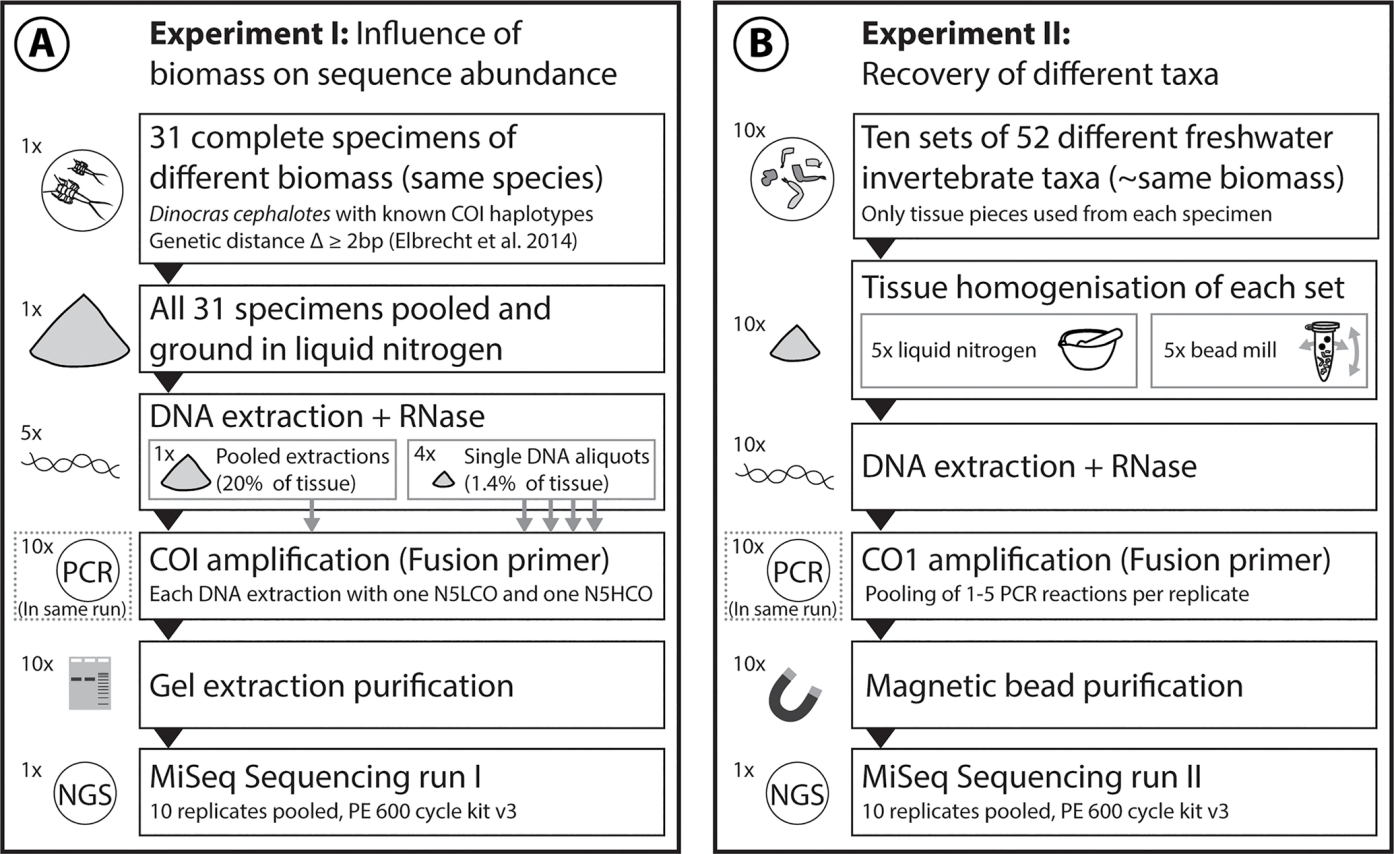
Overview of the experimental setup of Experiment I and II. Two MiSeq runs were used to increase the reproducibility and reliability of our novel metabarcoding protocol. **A** Experiment I: *Dinocras cephalotes* specimens with different COI barcodes were used to determine the reproducibility of the protocol and the influence of biomass on sequence abundance. **B** Experiment II: Ten sets of 52 aquatic taxa were homogenised, and DNA was extracted and amplified to determine which taxa could be recovered with MiSeq using the "ready to load" primers developed in this study.

Two experiments were performed (Fig 1). In experiment I, the influence of biomass on sequence abundance and the reproducibility of the method were tested using 31 stonefly specimens of the same species (*Dinocras cephalotes*), i.e., standardizing for a single species. In experiment II, species detection rates were tested using the standard barcoding primers LCO1490 and HCO2198 [41] and controlling for tissue biomass.

Ethics statement: No protected species and areas were sampled for this study with the exception of the dragonfly larvae *Cordulegaster* sampled from the Deilbach (N51.3282, E7.1619). Here, special permissions were obtained beforehand from the Kreisverwaltung Ennepe-Ruhr and Mettmann. No further permissions were required for sampling all other non-protected species from the Felderbach (N51.3450, E7.1703) and Ruhr University Bochum pond (N51.4457, E7.2656).

### Primer design and sequencing strategy

Fusion primers were designed that combined the standard COI LCO1490 (LCO) and HCO2198 (HCO) [41] with Illumina sequencing tags (S1 Fig). The advantage of fusion primers is that the COI barcoding fragments can be loaded directly onto the MiSeq sequencer after a single PCR and a purification step. COI amplicons are typically similar in base composition; therefore, three strategies were used to increase sequence diversity. First, 20% PhiX control was spiked into both MiSeq sequencing libraries. The PhiX library consists of fragments of a whole viral genome, which has a high nucleotide diversity. Second, the bases before the start of the Folmer primers were shifted by 0–4 bp increasing nucleotide diversity of amplicons at each read position (as described in [42]). Finally, a new approach was developed to increase diversity by simultaneously sequencing both LCO and HCO primers. A much higher per-site nucleotide diversity was observed using the consensus sequences of the 31 unique *Dinocras cephalotes* COI haplotypes (see experiment I below) (S2 Fig). Fewer peaks of low diversity (only up to 70% identical bases) were detected using both primers than using one primer at a time (100% identical bases). The parallel sequencing approach thus substantially reduced regions of low per-site diversity, improving read quality.

The adopted strategy of sequencing with LCO and HCO primers simultaneously as well as the 4-bp shifting strategy were used to differentially tag each of the ten replicates of both sequencing runs, allowing the removal of the Illumina tag in the adapters and the omission of the tag-reading step in the MiSeq runs.

### Experiment I: Relationship between specimen biomass and sequence abundance

#### Samples and DNA extraction

*D*. *cephalotes* larvae with known COI haplotypes were available from a previous study [43]. All specimens were stored in 96% ethanol at -20°C. From the specimens, 31 samples with different biomasses that differed by at least 2 bp in the COI barcode from all other specimens were selected (for GenBank accession numbers see S1 Table). All specimens were photographed and one leg was removed as a backup. All specimens were dried overnight at room temperature, weighed with a Sartorius RC 210D scale (0.01 mg accuracy) by two scientists independently and mean values were used for subsequent analyses. For bulk DNA extraction, the 31 specimens (with a cumulative weight of 642.72 mg) were placed in a ceramic mortar and manually ground into a fine powder (20 min processing time) using liquid nitrogen (Fig 1A). One-fifth of the ground tissue was divided among 14 reaction tubes at ~9 mg each (9/642.72 = 1.4% of total tissue). DNA was extracted from the 14 aliquots using a modified salt extraction protocol [44]. Extraction success was checked on an agarose gel. Then, 25 μL of DNA from each aliquot was treated with 0.55 μL of RNase (concentration 10 mg/mL, Thermo Scientific, Waltham, MA, USA) at 37°C for 30 min and cleaned up using the MinElute Reaction Clean up Kit to remove RNA (Qiagen, Hilden, Germany). The DNA concentration after cleanup was quantified using a Qubit 2.0 (Life Technologies, Carlsbad, CA, USA) with the Broad-Range (BR) Assay Kit.

#### COI amplification and sequencing

Four randomly chosen DNA aliquots (1.4% of total tissue) were selected for COI amplification. In addition, 50 ng DNA from each of the 14 DNA aliquots was pooled to create a sample representing 20% of the total tissue. For each of these five samples, two PCR were run using one N5LCO and one N5HCO fusion primer, each uniquely tagged (S1 Fig). The same PCR master mix was used for all reactions to ensure identical PCR conditions for all replicates. The ten PCR replicates were then run simultaneously in a C100 Thermalcycler (BioRad, Hercules, CA, USA).

The COI fragment was amplified in a PCR reaction consisting of 1× PCR buffer (including 2.5 mM Mg^2+^), 0.2 mM dNTPs, 0.5 μM of each primer, 0.025 U/μL of HotMaster Taq (5Prime, Gaithersburg, MD, USA), 50 ng DNA, and HPLC H_2_O to a total volume of 50 μL. The PCR program was as follows: 94°C for 180 s, 30 cycles of 94°C for 30 s, 46°C for 30 s, and 65°C for 150 s, and 65°C for 5 min. PCR products were excised from a 1% TAE agarose gel and purified using the MinElute Gel Extraction Kit (Qiagen, Hilden, Germany). Concentrations were measured using the Qubit 2.0 BR Kit and the library for sequencing was prepared by pooling 12.3 ng of all ten amplicons. Then, paired-end sequencing was carried out by GATC Biotech (Constance, Germany) using the MiSeq with 300 bp paired-end sequencing.

#### Bioinformatic analysis

S3A Fig includes a flow chart of the data processing steps. Sequences with a Phred score of >20 were demultiplexed using the base shift tags in both read directions using an R script (available on request). Primers were removed with cutadapt 1.4.2 [45] and forward and reverse reads were concatenated to 540-bp fragments. Paired end sequencing generated 2*300 bp long fragments, which is not enough to recover the complete Folmer COI region, which is typically 658 bp in length. Furthermore, up to 30 bp of the reads are primer sequences, leading to 2*270 = 540 bp concatenated fragments. Sequences of each replicate were compared against a reference database using the blastn algorithm (blastn 2.2.29, [46]). Statistics and data subsetting were performed in R 3.1.2 [47]. Hits shorter than 500 bp and those that matched two haplotypes equally well owing to sequencing errors and chimeras were removed from the hit table. To ensure reliable hits, only sequences that had a maximum of five mismatches and gaps were included in the analysis. The number of hits per haplotype was calculated and compared to the weight of the corresponding specimens.

### Experiment II: Recovery of 52 different taxa

#### Samples and DNA extraction

Freshwater macroinvertebrates were collected from various streams (and *Daphnia* from ponds) in western Germany and stored in 96% ethanol. Specimens were identified to the lowest taxonomic level possible based on morphology. Ten sets consisting of 52 unique taxa were photographed, and roughly equal amounts of tissue were dried overnight and weighed (Fig 1B, S2 Table). For *Isoperla*, Limoniidae, Tipulidae, and *Cordulegaster boltonii*, fewer than ten specimens were obtained; therefore, tissue from a single specimen was used in more than one replicate extraction. As each distinct morphotaxon was present only once in each of the ten replicates, barcoding prior to DNA extraction was not necessary. The 52 tissue samples per replicate were pooled for DNA extraction, and five replicates were ground in liquid nitrogen for 20 min, while the other five replicates were ground with a Qiagen TissueLyser LT (two times for 2 min at 50 Hz with a short centrifugation of the tubes in between). DNA was then extracted with the salt extraction protocol described in experiment I, and 10 μL of DNA for each of the 14 extraction tubes was pooled for each of the ten replicates. RNA was digested prior to PCR as described in experiment I.

#### COI amplification and sequencing

The PCR conditions were identical to those used in experiment I, and all ten replicates were run simultaneously in a C100 Thermalcycler (BioRad, Hercules, CA, USA) using the same master mix (see above). One to five PCR aliquots were pooled for each of the ten replicates with the aim to test whether replication of PCR reduces stochastic effects (was not evaluated, due to small number of replicates). Amplicons were purified and size selected (500–1000 bp) using magnetic beads (SPRIselect, Beckman Coulter, Bread, CA, USA; ratio 0.55×/0.45×). PCR product concentrations were measured using the Qubit BR Kit and the library for sequencing was prepared by pooling 52 ng of all ten replicates. 300 bp paired-end sequencing on a MiSeq was performed by GATC Biotech.

#### Bioinformatic analysis

S3B Fig includes a flow chart of the data processing steps. Reads were demultiplexed with a minimum Phred score of 25, primers were removed, and reads were concatenated as described in experiment I. Sequences from all ten replicates were pooled and dereplicated, and singletons were removed to find operational taxonomic units (OTUs) using the UPARSE pipeline (97% identity, [37]). Chimeras were removed from the OTUs using uchime_denovo. The remaining OTUs were identified using the BOLD barcoding database by querying against all barcode records. The ten replicates were dereplicated using derep_fulllength, but singletons were included in the data set. Sequences were matched against the OTUs with a minimum match of 97% using usearch_global. The hit tables were imported and the sequence numbers were normalised to the total sequence abundance and tissue weight for the various taxa.

## Results

### Sequencing success and statistics

The MiSeq runs of experiments I and II yielded 9.63 and 14.07 Gb of read data, respectively. Both MiSeq runs showed good read quality (sequences with Q30 ≥ 85.3% and 83.3%). The complete MiSeq data from both runs are available online on NCBI with the accession numbers SRS731403 (experiment I) and SRS733820 (experiment II).

Sequences starting with the LCO primer for the first read were significantly overrepresented in experiment I (34% more frequent than sequences beginning with the HCO primer, *t*-test, p = 0.006, S4A Fig). In experiment II, we did not detect primer bias (*t*-test, p = 0.41, S4B Fig). However, each of the ten replicates had a unique tissue set, in contrast to experiment I, which used the same DNA pool derived from a single species (31 specimens) for all five aliquots.

When normalising the loss of sequences in each data processing step of experiments I and II, we found significant differences between the LCO and HCO primers with respect to the number of reads with no hits in experiment I (*t*-test, p = 0.01, S3A Fig) and final hits in experiment II (*t*-test, p = 0.01, S3B Fig). The primers (LCO and HCO) had small effects on final sequence abundance, with differences of 2.7% and 4.4% for experiment I and II. We observed a similarly small effect on sequences abundance for the individual specimens in experiment I; there were significant differences in sequence abundance between LCO and HCO for 17 of the 31 stonefly specimens (*t*-test, p<0.05, S5 Fig).

### Experiment I

#### Amount of extracted tissue and species recovery

**Fig 2:**
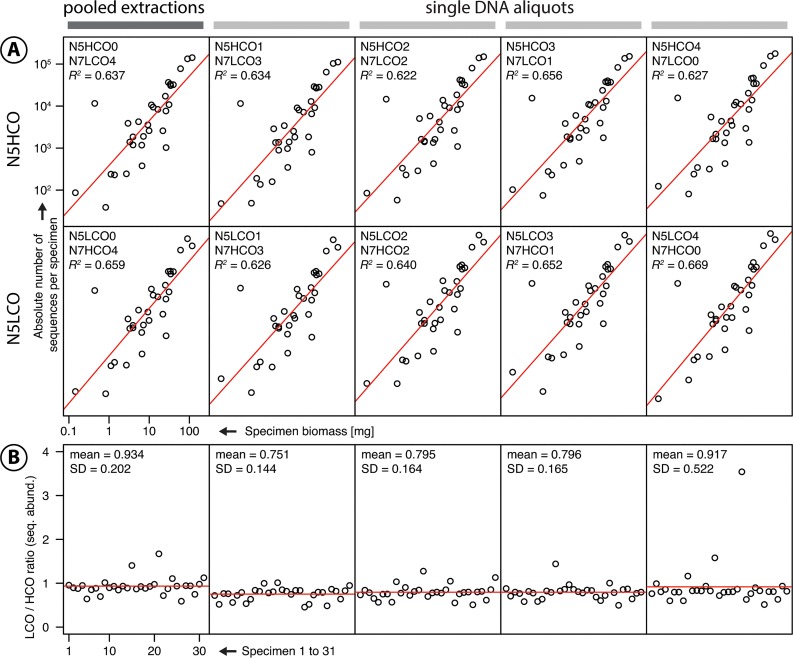
Results of Experiment I: Dependence of read abundance on specimen biomass. **A** Specimen weight (*y*-axis) is plotted against read abundance per specimen (*x*-axis) for all ten replicates. The linear regression (red line) was highly significant for all replicates with p<0.001. **B** Ratio of N5LCO/N5HCO sequence abundance for the mixed and four single DNA aliquots. The red line indicates the mean ratio.

S1 Table gives an overview of the weights of the 31 specimens. The two independent weight measurements differed in mean by 0.1 mg (SD = 0.03). In all 10 replicates, we recovered all 31 *D*. *cephalotes* specimens based on their unique haplotypes, including the smallest specimens, which only made up 0.023% (0.145 mg) of the total specimen biomass (Fig 2A). For two specimens, we recovered sequence artefacts (see S6 Fig); these did not affect further analyses. We did not observe significant differences in sequence abundance among the replicates using different amounts of extracted tissue as a template. However, there was slightly more variation in sequence abundance for the replicates in which DNA was extracted from only 1.4% of the total tissue volume than from 20% (S7A Fig). We observed a strong negative relationship between specimen weight and variation in sequence abundance, as shown in S5 and S7B Figs. Specimens with a low biomass tend to show relatively high variation in sequence abundance among the ten replicates.

#### Influence of biomass on sequence abundance

We found a highly significant positive linear correlation between specimen biomass and sequence abundance in all ten replicates (Fig 2A). The mean normalised sequence abundances had low standard deviations and the linear model fit well (p<0.001, *R* ^2^ = 0.65, S8 Fig).

#### Reproducibility of sequencing results

The sequencing results for the ten replicates were highly reproducible. Even when comparing absolute sequence numbers, the patterns were concordant (Fig 2A). We detected few outliers and low standard deviations for the ratio of LCO- to HCO-based haplotype read abundance for each of the five DNA extractions (Fig 2B and S8 Fig).

### Experiment II

#### Recovery of different taxa with similar biomass

**Fig 3:**
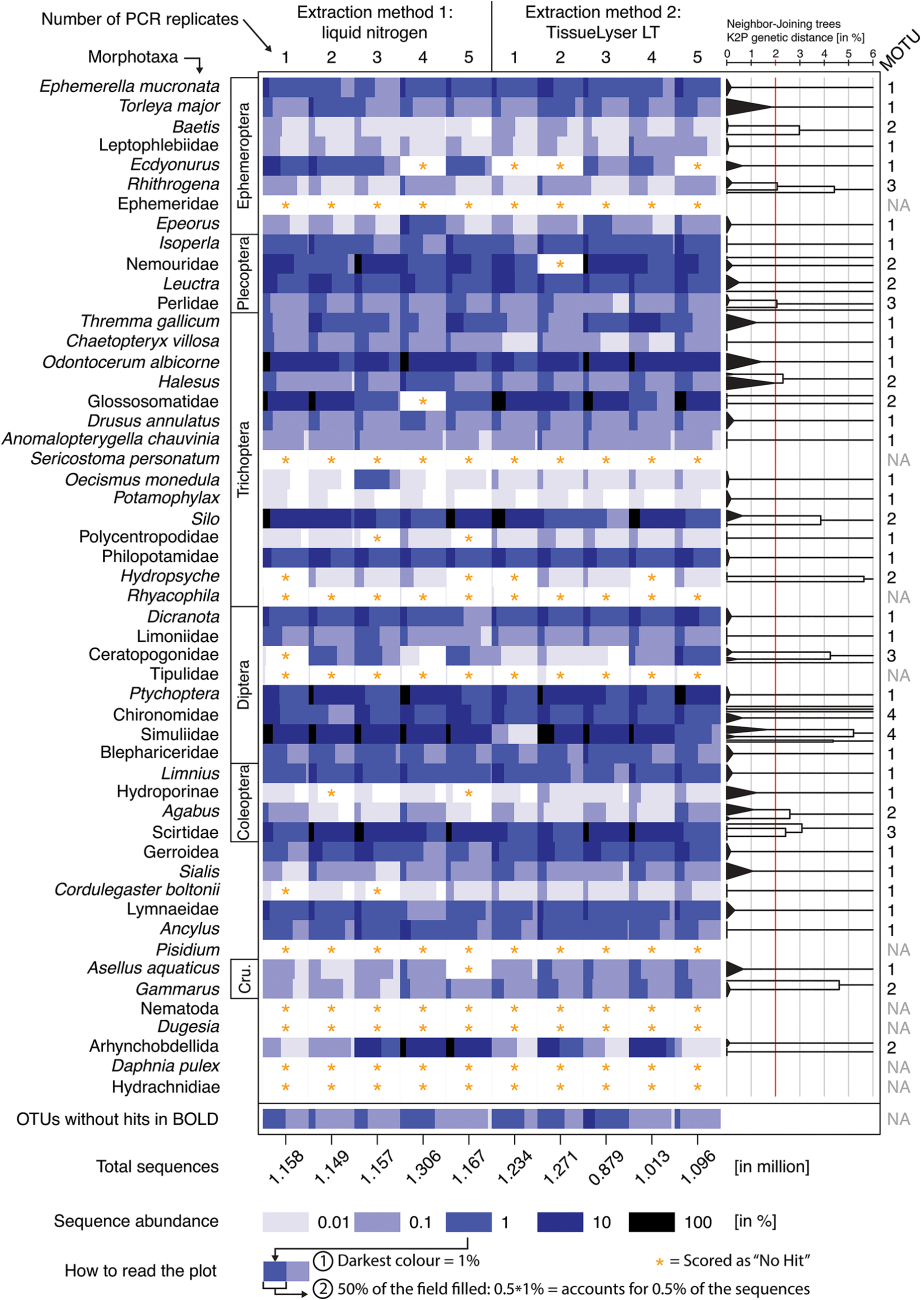
Overview of taxa recovery in experiment II. Sequence abundances for the 52 morphologically identified taxa is shown in rows and the ten replicates used in the experiment in columns. Sequence abundance was normalised across the ten replicates and the amount of tissue used in each extraction. Sequence abundance of each specimens (morphotaxon) of the ten replicates is visualised by different shades of blue. If a field is i.e. half filled (50%) with the mid blue shade (= 1% of total sequences), the respective specimen represented 0.5% (50% of 1%) of the total sequences in that replicate. When no sequences or only a few sequences (below 0.003% of total abundance per replicate) were found for a specimen, it was scored as "No Hit," as indicated by an orange asterisk. On the right, K2P-corrected neighbour-joining (NJ) trees for each taxon, based on the most abundant sequence obtained for each specimen (calculated with MEGA6.06), are shown. MOTUs are defined by a 2% sequence difference based on the NJ tree.

S2 Table gives an overview of specimen weights of the 52 tissues parts, used in each of the ten replicates. We were able to reliably recover 83% (43) of the 52 taxa included in experiment II. We recovered many of the typical bio-indicator taxa such Ephemeroptera, Plecoptera, Trichoptera, and Diptera (Table 1). 34 taxa were recovered in all ten replicates (Fig 3). From the DNA extractions performed with the TissueLyser LT, six more specimens (2.31%) were recovered than when DNA was extracted with liquid nitrogen. Furthermore, we did not observe substantial differences in recovery rates when different numbers of PCR products were pooled.

**Table 1:** Number of specimens recovered (for four major taxonomic groups and “others”) in experiment II.

Taxonomic group	Recovered specimens	
Ephemeroptera	7/8	(88%)
Plecoptera	4/4	(100%)
Trichoptera	13/15	(86%)
Diptera	7/8	(88%)
Others	12/17	(71%)
Σ Taxa	43/52	(83%)

On average, 99.52% of the hits could be assigned to the specimens used in each extraction (Fig 3 and S9A Fig). Sequences that did not match the target species were similar to sequences derived from a variety of benthic organisms, but also fungi and plants. Of all recovered 213 OTUs, 31 could not be identified using the BOLD database (S9B Fig).

#### Higher taxonomic resolution

We were able to reliably assign OTUs to all 52 taxa included in the extraction. In 19 of the cases (37%), the morphologically identified taxa were assigned to more than one MOTU (K2P distance of detected haplotypes > 2%, Fig 3), indicating that the ten morphologically identified specimens per morphotaxon included several species. For example, we found two distinct MOTUs in the caddisfly genus *Silo*, three in the mayfly genus *Rhitrogena*, and four in the blackflies (Simuliidae) (Fig 3). This was expected, as morphotaxa could often only be identified at family or order level, and several species per morphotaxon may occur in the sampled aquatic habitats even at the same habitat patch. However, most morphotaxa represented a single MOTU (i.e. distinct biological species). Identification by COI barcode did not perform worse than identification by morphology. In fact, in 50% of the cases, barcoding identified specimens at a finer taxonomic level, and 11 morphotaxa included multiple species using COI data from the BOLD database (S9A Fig).

#### Variation of sequence abundance for different taxa

Although many taxa were recovered, the number of sequences per taxon varied by four orders of magnitude despite the similarity in biomass used for extraction (Fig 3). In most cases, the sequence abundance obtained for a certain morphotaxon was consistent across replicates after normalising for slight differences in the amount of tissue biomass used. Exceptions were Arhynchobdellida, Ceratopogonidae, Scirtidae and Glossosomatidae (S3 Table), where efficiencies differed substantially between the MOTUs. There was no correlation between taxon biomass and the number of recovered sequences (using the means of ten replicates for each of the 52 taxa).

## Discussion

Several studies show that DNA-based assessments are superior to morphological assessments in freshwater ecosystems (e.g., [14,25,33]); yet, reliable and standardised laboratory protocols need to be established prior to integrating metabarcoding assessments into existing monitoring programs. Here, we developed a new laboratory workflow and generated highly replicated next-generation sequencing data using the traditional 658-bp Folmer fragment. We used this data to systematically test, for the first time, for a relationship between specimen biomass and sequence reads in a standardised single-species setting (experiment I), and then assessed the impact of primer bias for multi-species samples using standardised biomass pools (experiment II).

### Experiment I: Relationship between specimen biomass and sequence abundance

Benthic indicator organisms vary in biomass, and this variation depends on taxonomic group and life stage. Therefore, it is crucial to determine the relationship between biomass and sequence abundance to i) estimate taxon biomass in samples from read data, and ii) identify critical detection limits for a given sequence coverage and sample size. Piñol et al. [39] proposed a relationship between biomass and sequence reads, but did not systematically examine this hypothesis. Using 31 specimens of a single stonefly species (see [43]) that differed in biomass, we demonstrated a highly significant correlation between sequence abundance and specimen biomass. Irrespective of the tissue volume used for extraction, we recovered all 31 specimens. Weak outliers might be caused by differences in tissue conservation or mismatches in the primer binding sites of individual specimens [48]. Variation in sequence abundance for each specimen was slightly higher among replicates that had less tissue as starting material. This trend was stronger for specimens with a smaller biomass. This result is expected because stochastic effects increase with reduced specimen biomass. However, our results indicate that the amount of tissue used in the DNA extraction was sufficient and did not lead to the systematic exclusion of small specimens as long as tissues are well ground and specimens have similar amplification efficiencies.

Furthermore, the LCO and HCO PCR replicates for DNA extraction yielded highly concordant results, emphasising the overall reliability of our protocol. We detected a slightly different sequencing efficiency between forward and reverse primers, but this only affected total sequence abundance and did not systematically alter the inferences from the data.

### Experiment II: Primer amplification bias between species

Amplification of diverse multitemplate mixtures using universal primers can lead to highly unequal amplification efficiencies among products [39,49]. It has therefore been suggested that several group-specific primers are necessary for species monitoring [32]. Here, we quantified the effect of primer bias using similar tissue biomass and different species (using ten replicates) to determine whether reliable species detection is possible.

The number of sequences per replicate was not biased by primer type (LCO or HCO), indicating that sequencing direction has a negligible effect when independent replicates are sequenced. Furthermore, we recovered a majority of specimens (83%) using one universal primer pair. However, using the Folmer primers, the number of sequences obtained varied among taxa by several orders of magnitude, probably because there were mismatches in the primer binding regions.

While amplification efficiencies were consistent among replicates of a morphotaxon, in particular when determined to species or genus level, some taxa that could only be determined at higher taxonomic level, contained different MOTUs with different amplification efficiencies (e.g. the order Arhynchobdellida). Presumably, these morphotaxa contained several taxonomically distant species, which are unequally well amplified with the Folmer primers. This is consistent with the primer bias which we already observed between morphotaxa and support the overall findings of strong taxon-specific amplification bias.

### Implications for large-scale monitoring and future challenges

In this study, we established a quick and reliable protocol to assess the macrozoobenthic communities of stream ecosystems. We used a highly replicated and standardised approach for species detection using DNA metabarcoding and show that several technical and logistic problems have to be overcome before this protocol can be used for large-scale monitoring.

The results of experiment I show that it is possible to reliably estimate the biomass of a single species, but not its abundance because many small organisms generate the same number of sequence reads as a few large organisms. However, the results of experiment II show that primer efficiencies across different taxa greatly hinder species abundance assessments using PCR-based approaches, which is consistent with the findings of Piñol et al. [39]. Thus, it is not possible to accurately estimate species biomass or even abundance in diverse environmental samples using amplification-based sequencing protocols. For accurate estimates of biomass, or even rough estimates, a PCR-free approach is needed; however, this requires further development [50]. Currently, the monitoring of freshwater ecosystems is based on abundance metrics, which cannot be generated using the metabarcoding solutions currently available. Thus, for now, monitoring indices should use genetic data for presence-absence assessments. Initial studies on marine benthic taxa show that presence-absence data is sufficient for precise assessment indices [51,52], especially considering the additional information gained by species-level identification. Thus, the availability of such highly reliable data on the presence of species (even cryptic) can be very important for community descriptions. Furthermore, we used a universal COI primer with a broad target range [41], and only 17% of taxa went undetected. While this is already better than the error rates of several morphology-based studies (see [10] for a discussion), higher detection rates are desirable. This could be achieved if several group-specific primers or even more degenerate primers are used [32]. Our protocol uses ten tagged fusion primers, and the Folmer primers can easily be supplemented with group-specific primers. To ensure that small specimens are detected, samples can additionally be fractionated into various size categories; extractions can be performed independently for each category, and template DNA amounts can be adjusted according to specimen size prior to amplification and sequencing.

The methods developed in this study can easily be adapted to assess the communities of other ecosystems. Our parallel sequencing strategy leads to an increase in per-site sequence diversity and read quality. The approach can be easily integrated into any other protocol for the MiSeq, HiSeq, or NextSeq protocols. While the use of full COI barcodes targeting the classical primer regions might give the highest taxonomic resolution, mini barcodes might be sufficient to detect most species and are popular in environmental DNA barcoding [53]. However degraded DNA and contamination as in eDNA studies with amplicons is not a concern for organisms collected directly from streams. An approximately 400-bp barcode lying within the standard Folmer region would be optimal for both strategies, and plenty of sequence information is available to develop group-specific primers [29,32]. The use of a single universal primer pair that amplifies conserved ribosomal mitochondrial gene regions (e.g., 16S and 12S) could be effective [54]. However, while this approach could have comparable taxonomic resolution as the COI barcode, it is currently limited by the lack of reference databases [55].

All assessment protocols rely on reference catalogues against which inventory data of a species from an ecosystem are compared. In particular, changes in species traits in a community (e.g. functional feeding groups, tolerance against pollution) are used as indicator values to evaluate the biological significance of inferred community changes. The efforts of large national and international barcoding consortia (BOLD, iBOL, and GBOL) have contributed to a substantial increase in both the size and quality of reference databases [56], which has provided a basis for species-level assessments. The protocol developed in this study enables the identification of nearly all macrozoobenthic species in an environmental sample. However, DNA-based assessments cannot assign biological traits to species. Therefore, to take full advantage of metabarcoding, acquiring ecological trait data at the species or even population level is the next crucial step. A combination of both data types, i.e., DNA species barcodes and ecological traits, will maximise the power of metabarcoding for the reliable assessment of ecosystem responses under stress and for biomonitoring.

Although more technical developments are necessary, we are confident that metabarcoding will widely replace present biomonitoring methods over time because 1) it has a higher taxonomic resolution [14], 2) it is cost efficient and fast and, most importantly, 3) it reduces human bias enabling comparisons among studies [57]. Using our protocol, it is possible to assess community compositions within a week, from sampling to species identification.

## Conclusions

We provide a highly reproducible laboratory protocol for processing macroinvertebrate samples in bulk and identified species using metabarcoding with the standard COI region. The technical accuracy of this method was supported by comparisons among many replicates. However, we also showed that the taxon abundance of diverse environmental samples can not be reliably assessed. Therefore, we suggest focusing on reliable presence-absence data obtained from replicated analyses. We are confident that the here presented protocol could be a useful resource to monitor a wide range of ecosystems in the next years.

## Supporting Information

S1 Fig Fusion COI Primers developed in this study. Fusion primer can be directly loaded onto the MiSeq system and universal primers modified or replaced.
(PDF)

S2 Fig Increase of diversity by parallel sequencing. By sequencing forward and reverse primers together, sequence diversity and thus read quality is increased.
(PDF)

S3 Fig Number of reads excluded in data processing steps. Includes flow charts of the bioinformatics processing of experiment I (A) and experiment II (B).
(PDF)

S4 Fig Reads in each replicate after demultiplexing. Data from experiment I (A) and experiment II (B).
(PDF)

S5 Fig Experiment I: sequences per specimen. Normalised sequence abundance for each stonefly.
(PDF)

S6 Fig Experiment I: sequencing artefacts. Sequence matches are shown for three individual specimens, including h28 and h13 that are affected by sequencing artefacts.
(PDF)

S7 Fig Experiment I: Variability in sequence abundance. Variability in sequence abundance between the ten replicates as well as dependence on specimen biomass.
(PDF)

S8 Fig Experiment I: Sequence abundance depended on specimen biomass. Mean normalised sequence abundance of all ten replicates, including standard errors.
(PDF)

S9 Fig Experiment II: OTUs assigned to taxa. Detailed overview of all 213 OTUs and their taxonomic identification using the BOLD database.
(PDF)

S1 Table Information on *Dinocras cephalotes* specimen weights (in milligram) for experiment I (XLS)

S2 Table Information on specimen weights (in milligram) for experiment II. (XLS)

S3 Table MOTU assignment to individual specimens in experiment II. (XLS)
