# Supplementary material for: Can DNA-Based Ecosystem Assessments Quantify Species Abundance? Testing Primer Bias and Biomass—Sequence Relationships with an Innovative Metabarcoding Protocol
Source: PLoS One. 2015 Jul 8;10(7):e0130324. doi: 10.1371/journal.pone.0130324 (PMC4496048; doi:10.1371/journal.pone.0130324)
Supplement: S1 Fig — Fusion primer can be directly loaded onto the MiSeq system and universal primers modified or replaced. (PDF) [file pone.0130324.s001.pdf]

N5HCO0 AATGATACGGCGACCACCGAGATCTACACTCTTTCCCTACACGACGCTCTTCCGATCTTAAACTTCAGGGTGACCAAAAAATCA
N5HCO1 AATGATACGGCGACCACCGAGATCTACACTCTTTCCCTACACGACGCTCTTCCGATCTCTAAACTTCAGGGTGACCAAAAAATCA
N5HCO2 AATGATACGGCGACCACCGAGATCTACACTCTTTCCCTACACGACGCTCTTCCGATCTGATAAACTTCAGGGTGACCAAAAAATCA
N5HCO3 AATGATACGGCGACCACCGAGATCTACACTCTTTCCCTACACGACGCTCTTCCGATCTTGCTAAACTTCAGGGTGACCAAAAAATCA
N5HCO4 AATGATACGGCGACCACCGAGATCTACACTCTTTCCCTACACGACGCTCTTCCGATCTACGCTAAACTTCAGGGTGACCAAAAAATCA
N5LCO0 AATGATACGGCGACCACCGAGATCTACACTCTTTCCCTACACGACGCTCTTCCGATCTGGTCAACAAATCATAAAGATATTGG
N5LCO1 AATGATACGGCGACCACCGAGATCTACACTCTTTCCCTACACGACGCTCTTCCGATCTAGGTCAACAAATCATAAAGATATTGG
N5LCO2 AATGATACGGCGACCACCGAGATCTACACTCTTTCCCTACACGACGCTCTTCCGATCTTCGGTCAACAAATCATAAAGATATTGG
N5LCO3 AATGATACGGCGACCACCGAGATCTACACTCTTTCCCTACACGACGCTCTTCCGATCTCACGGTCAACAAATCATAAAGATATTGG
N5LCO4 AATGATACGGCGACCACCGAGATCTACACTCTTTCCCTACACGACGCTCTTCCGATCTCTACGGTCAACAAATCATAAAGATATTGG

flow cell bind N5 — sequencing primer 1 bind — HCO / LCO (Folmer et al. 1994)

N = shift (Lundberg et al. 2013)

N7LCO0 CAAGCAGAAGACGGCATACGAGATCGGTCTCGGCATTCCTGCTGAACCGCTCTTCCGATCTGGTCAACAAATCATAAAGATATTGG
N7LCO1 CAAGCAGAAGACGGCATACGAGATCGGTCTCGGCATTCCTGCTGAACCGCTCTTCCGATCTAGGTCAACAAATCATAAAGATATTGG
N7LCO2 CAAGCAGAAGACGGCATACGAGATCGGTCTCGGCATTCCTGCTGAACCGCTCTTCCGATCTTCGGTCAACAAATCATAAAGATATTGG
N7LCO3 CAAGCAGAAGACGGCATACGAGATCGGTCTCGGCATTCCTGCTGAACCGCTCTTCCGATCTCACGGTCAACAAATCATAAAGATATTGG
N7LCO4 CAAGCAGAAGACGGCATACGAGATCGGTCTCGGCATTCCTGCTGAACCGCTCTTCCGATCTCTACGGTCAACAAATCATAAAGATATTGG
N7HCO0 CAAGCAGAAGACGGCATACGAGATCGGTCTCGGCATTCCTGCTGAACCGCTCTTCCGATCTTAAACTTCAGGGTGACCAAAAAATCA
N7HCO1 CAAGCAGAAGACGGCATACGAGATCGGTCTCGGCATTCCTGCTGAACCGCTCTTCCGATCTCTAAACTTCAGGGTGACCAAAAAATCA
N7HCO2 CAAGCAGAAGACGGCATACGAGATCGGTCTCGGCATTCCTGCTGAACCGCTCTTCCGATCTGATAAACTTCAGGGTGACCAAAAAATCA
N7HCO3 CAAGCAGAAGACGGCATACGAGATCGGTCTCGGCATTCCTGCTGAACCGCTCTTCCGATCTTGCTAAACTTCAGGGTGACCAAAAAATCA
N7HCO4 CAAGCAGAAGACGGCATACGAGATCGGTCTCGGCATTCCTGCTGAACCGCTCTTCCGATCTACGCTAAACTTCAGGGTGACCAAAAAATCA

flow cell bind N7 — sequencing primer 2 bind — HCO / LCO (Folmer et al. 1994)

N = shift (Lundberg et al. 2013)

Figure S1. Fusion COI Primers developed in this study. The primers are based on the Folmer et al. (1994) primers. They include MiSeq flow cell and sequencing primer binding regions. All primers amplify an 832-bp fragment that can be sequenced directly after purification. Up to 10 libraries can be uniquely tagged and pooled in one MiSeq run with the tags consisting of the HCO or LCO primer + the primer shift up to 4 bp (always 4 bp in total). It is recommended that all 10 primer pairs are used in the following combination to maximise sequence diversity:

N5HCO0+N7LCO4, N5HCO1+N7LCO3, N5HCO2+N7LCO2, N5HCO3+N7LCO1, N5HCO4+N7LCO0,
N5LCO0+N7HCO4, N5LCO1+N7HCO3, N5LCO2+N7HCO2, N5LCO3+N7HCO1, N5LCO4+N7HCO0
