# Supplementary material for: Can DNA-Based Ecosystem Assessments Quantify Species Abundance? Testing Primer Bias and Biomass—Sequence Relationships with an Innovative Metabarcoding Protocol
Source: PLoS One. 2015 Jul 8;10(7):e0130324. doi: 10.1371/journal.pone.0130324 (PMC4496048; doi:10.1371/journal.pone.0130324)
Supplement: S2 Fig — By sequencing forward and reverse primers together, sequence diversity and thus read quality is increased. (PDF) [file pone.0130324.s002.pdf]

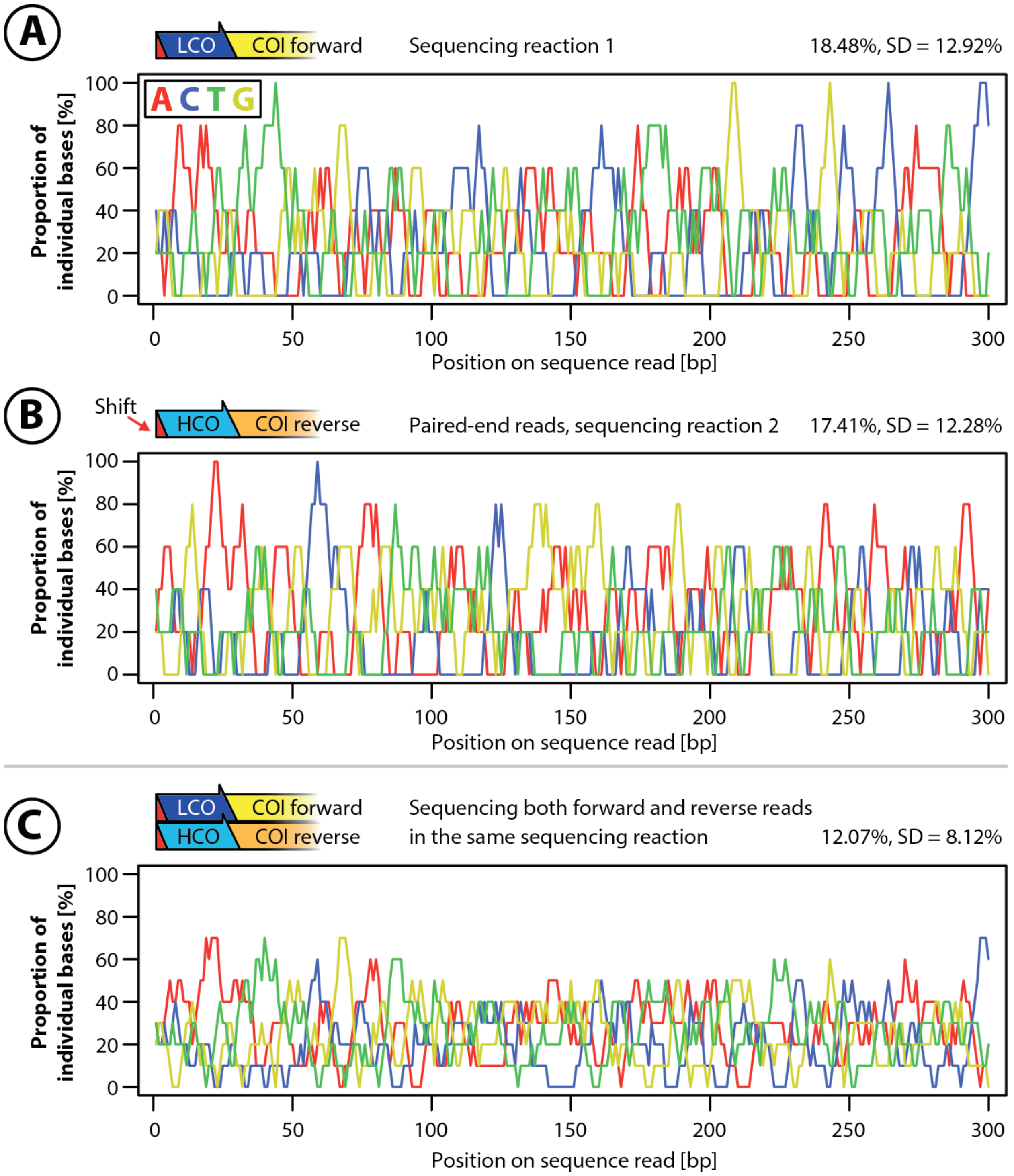

Figure S2. Estimated sequence diversity increase by parallel sequencing amplicons in LCO and HCO direction on the MiSeq. Estimated proportion of nucleotides per read position based on a majority consensus sequence of the 31 *Dinocras cephalotes* specimens using all 10 primer pairs developed in this study. The mean deviation from an ideal distribution of bases (each 25%) is indicated at the top right of each plot. **A** and **B** show the distribution of bases when LCO (read 1) and then HCO (read 2) is sequenced in the MiSeq run. In **C** both primers are sequenced in parallel in read 1 and read 2, which increases per-position diversity.
