# Supplementary material for: Can DNA-Based Ecosystem Assessments Quantify Species Abundance? Testing Primer Bias and Biomass—Sequence Relationships with an Innovative Metabarcoding Protocol
Source: PLoS One. 2015 Jul 8;10(7):e0130324. doi: 10.1371/journal.pone.0130324 (PMC4496048; doi:10.1371/journal.pone.0130324)
Supplement: S3 Fig — Includes flow charts of the bioinformatics processing of experiment I (A) and experiment II (B). (PDF) [file pone.0130324.s003.pdf]

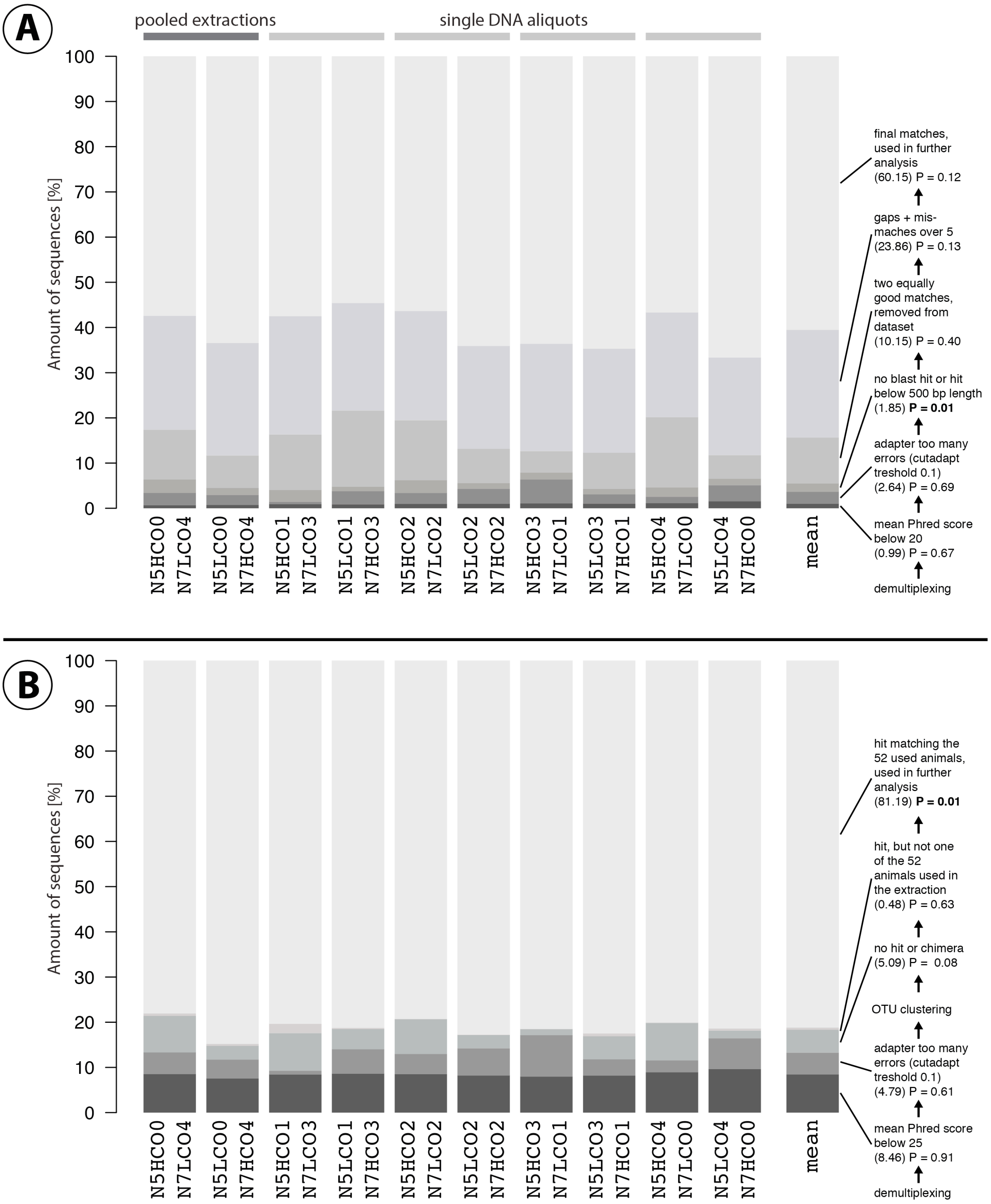

Figure S3. Number of reads excluded in data processing steps for experiment I (A) and experiment II (B). Mean sequence abundance in each processing step is written in brackets [in %]. N5HCO / N5LCO primer bias was tested with a *t*-test, and significant values are printed in bold.
