# Supplementary material for: Can DNA-Based Ecosystem Assessments Quantify Species Abundance? Testing Primer Bias and Biomass—Sequence Relationships with an Innovative Metabarcoding Protocol
Source: PLoS One. 2015 Jul 8;10(7):e0130324. doi: 10.1371/journal.pone.0130324 (PMC4496048; doi:10.1371/journal.pone.0130324)
Supplement: S4 Fig — Data from experiment I (A) and experiment II (B). (PDF) [file pone.0130324.s004.pdf]

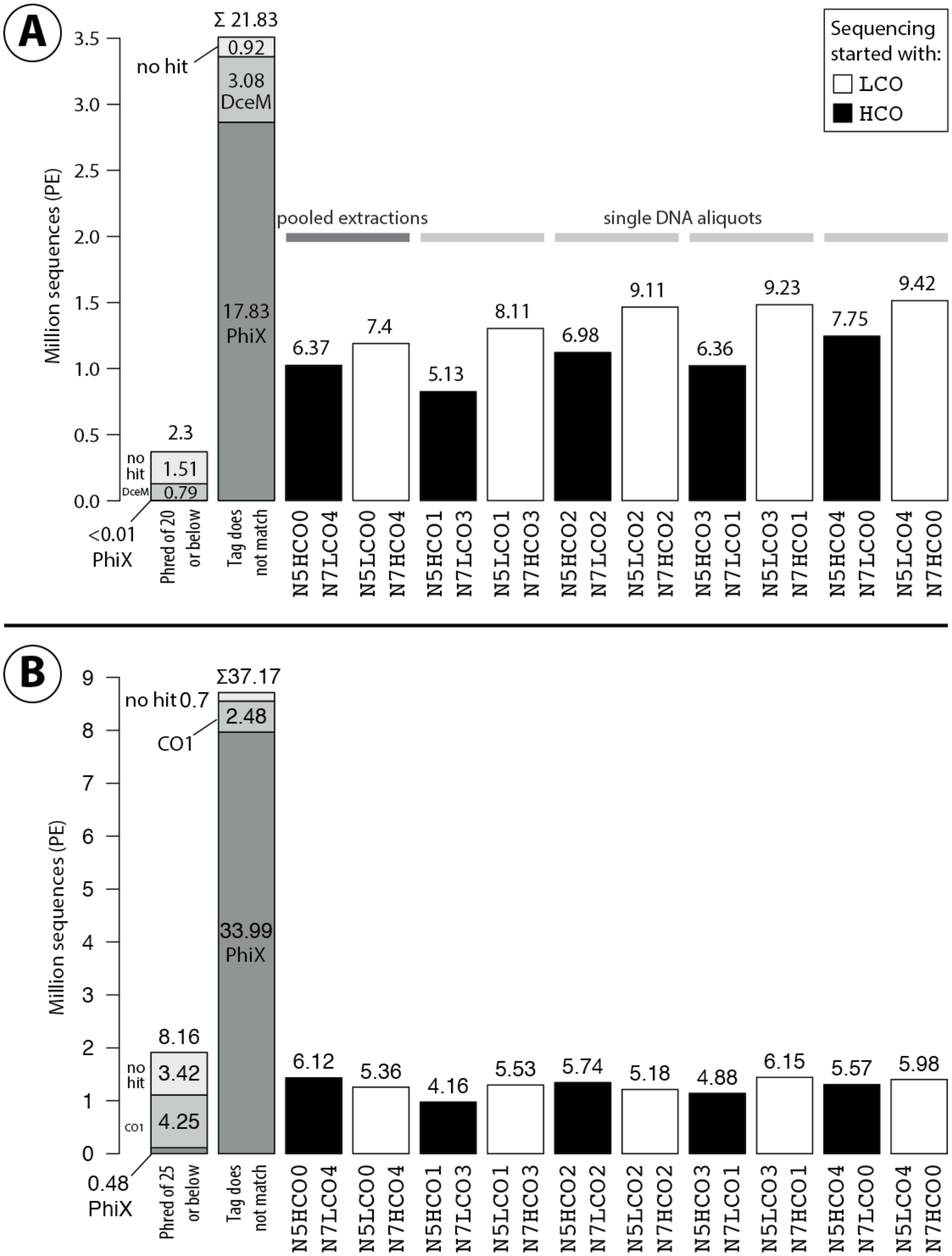

Figure S4. Number of sequences obtained in both experiments after demultiplexing. Numbers above bars indicate the relative contribution (in percent) to the total sequence number. If sequencing started with LCO (white) or HCO (black) is indicated by bar colour. A: Experiment I, amount of tissue used in respective DNA extraction indicated above the bars. B: Experiment II.
