# Supplementary material for: Can DNA-Based Ecosystem Assessments Quantify Species Abundance? Testing Primer Bias and Biomass—Sequence Relationships with an Innovative Metabarcoding Protocol
Source: PLoS One. 2015 Jul 8;10(7):e0130324. doi: 10.1371/journal.pone.0130324 (PMC4496048; doi:10.1371/journal.pone.0130324)
Supplement: S6 Fig — Sequence matches are shown for three individual specimens, including h28 and h13 that are affected by sequencing artefacts. (PDF) [file pone.0130324.s006.pdf]

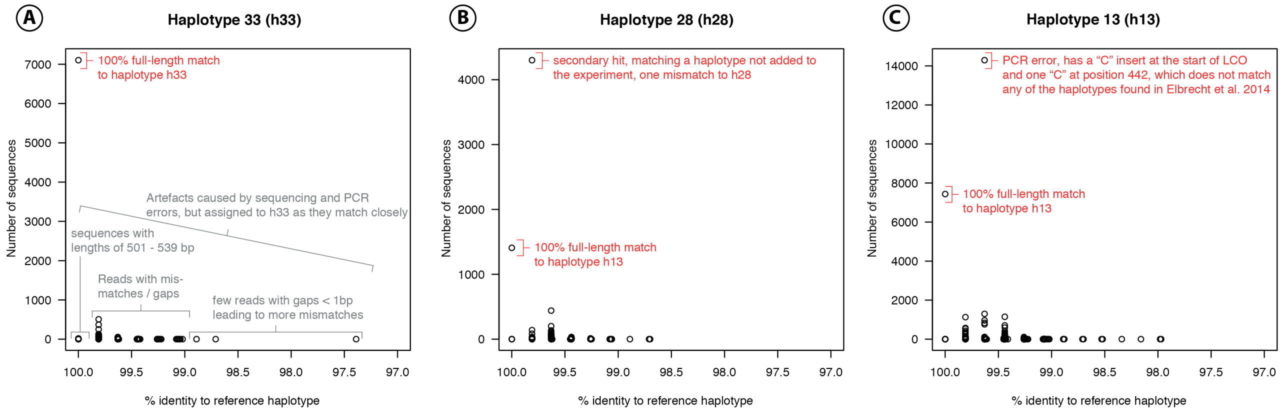

Figure S6. Experiment I: Abundance and percent match to reference sequence of individual haplotypes summarised for all 10 replicates. A Haplotype 33: representative pattern for 29 of 31 matches, one main peak 100% matching the reference haplotype and some closely matching sequences with up to five mismatches and gaps. **B** Haplotype 28: Abundant secondary hit, potentially caused by gut contents or tissue attached to a specimen (no evidence on pictures of specimens). **C** Haplotype 13: Secondary peak that is likely a PCR or sequencing error, as the C insert would break the reading frame and the haplotype shows a SNP not present in any of the samples in Elbrecht et al. (2014).
