# Supplementary material for: Can DNA-Based Ecosystem Assessments Quantify Species Abundance? Testing Primer Bias and Biomass—Sequence Relationships with an Innovative Metabarcoding Protocol
Source: PLoS One. 2015 Jul 8;10(7):e0130324. doi: 10.1371/journal.pone.0130324 (PMC4496048; doi:10.1371/journal.pone.0130324)
Supplement: S7 Fig — Variability in sequence abundance between the ten replicates as well as dependence on specimen biomass. (PDF) [file pone.0130324.s007.pdf]

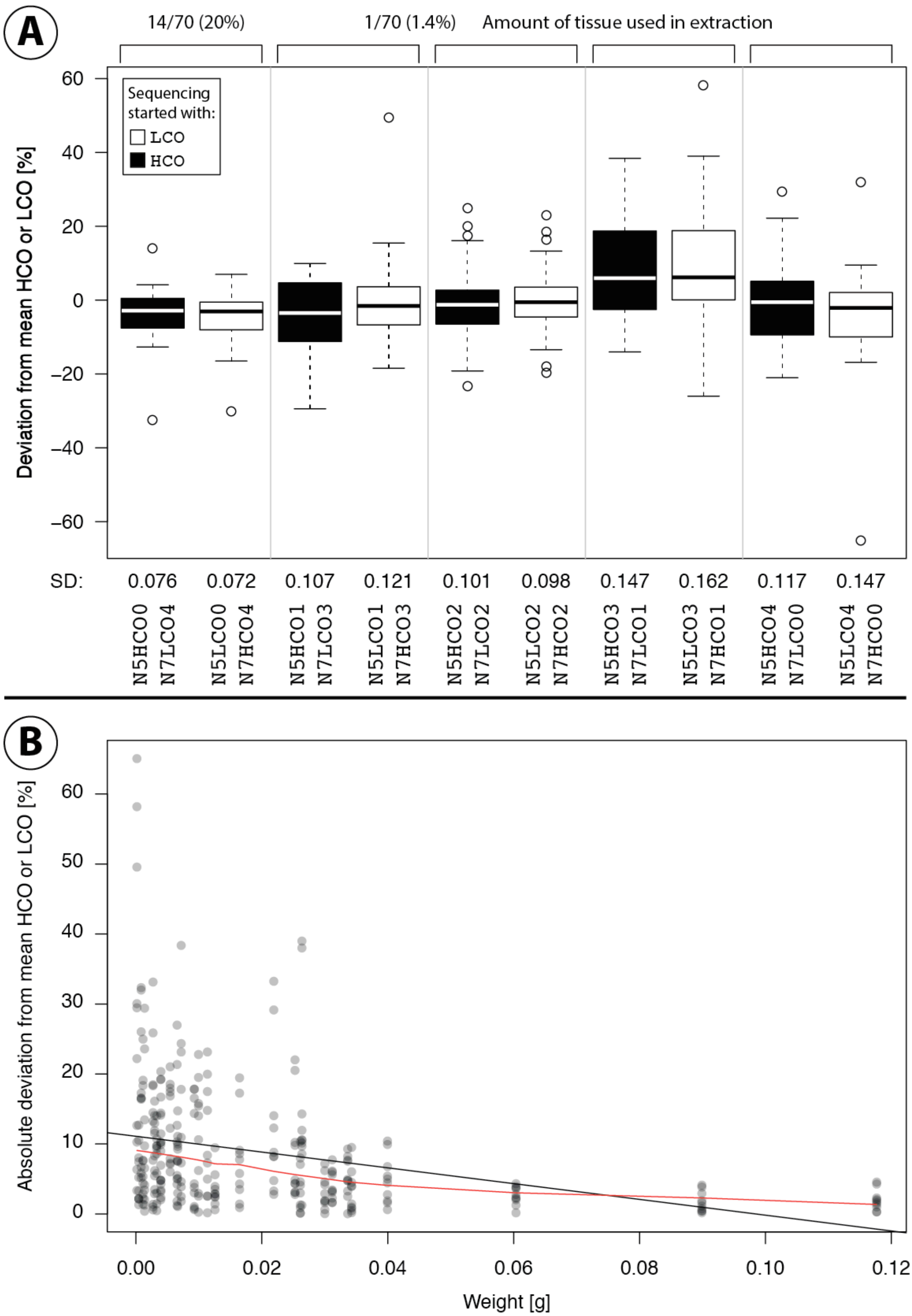

Figure S7. Experiment I: Variability of sequence abundance across the 10 replicates and dependence on specimen biomass. Calculated as deviation from normalised average sequence abundance for all haplotypes (calculated for the five LCO and five HCO separately due to primer bias). **A:** Variability of sequence abundance per specimen plotted for each of the 10 replicates. B: Variability of sequence abundance per specimen plotted against specimen weight for all 10 replicates (absolute values). Linear regression shown as black line ($P < 0.001$, $R^2 = 0.10$), red line shows LOWESS locally-weighted polynomial regression as implemented in R.
