# Supplementary material for: Can DNA-Based Ecosystem Assessments Quantify Species Abundance? Testing Primer Bias and Biomass—Sequence Relationships with an Innovative Metabarcoding Protocol
Source: PLoS One. 2015 Jul 8;10(7):e0130324. doi: 10.1371/journal.pone.0130324 (PMC4496048; doi:10.1371/journal.pone.0130324)
Supplement: S8 Fig — Mean normalised sequence abundance of all ten replicates, including standard errors. (PDF) [file pone.0130324.s008.pdf]

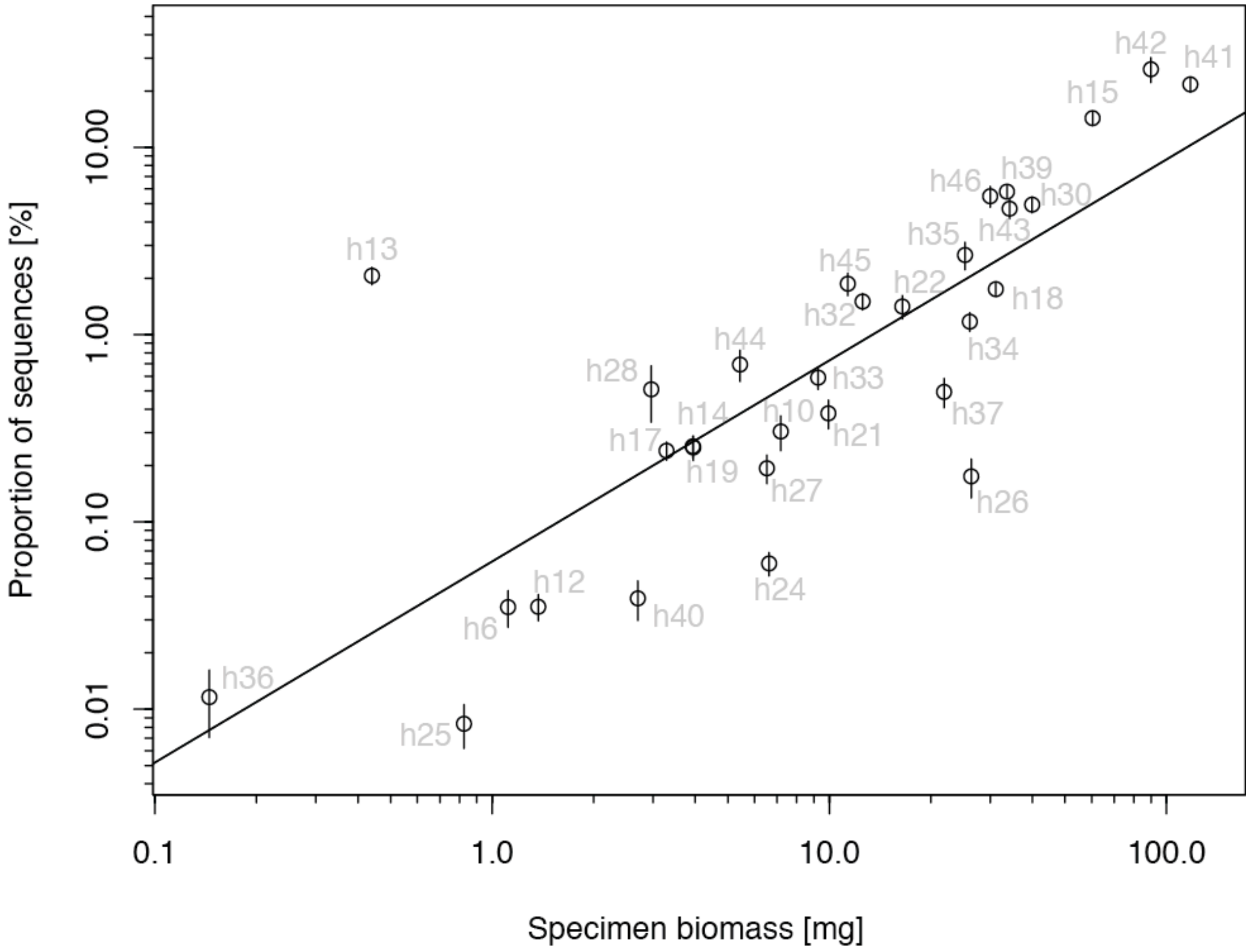

Figure S8. Experiment I: Dependence of specimen biomass on normalised sequence abundance. Sequence abundance is the mean of the 10 replicates, error bars represent standard deviations. The black line shows the linear regression ($R^2 = 0.65$, $p < 0.001$). The haplotypes of the 31 *Dinocras cepahlotes* specimens are indicated in grey.
