# Supplementary material for: Can DNA-Based Ecosystem Assessments Quantify Species Abundance? Testing Primer Bias and Biomass—Sequence Relationships with an Innovative Metabarcoding Protocol
Source: PLoS One. 2015 Jul 8;10(7):e0130324. doi: 10.1371/journal.pone.0130324 (PMC4496048; doi:10.1371/journal.pone.0130324)
Supplement: S9 Fig — Detailed overview of all 213 OTUs and their taxonomic identification using the BOLD database. (PDF) [file pone.0130324.s009.pdf]

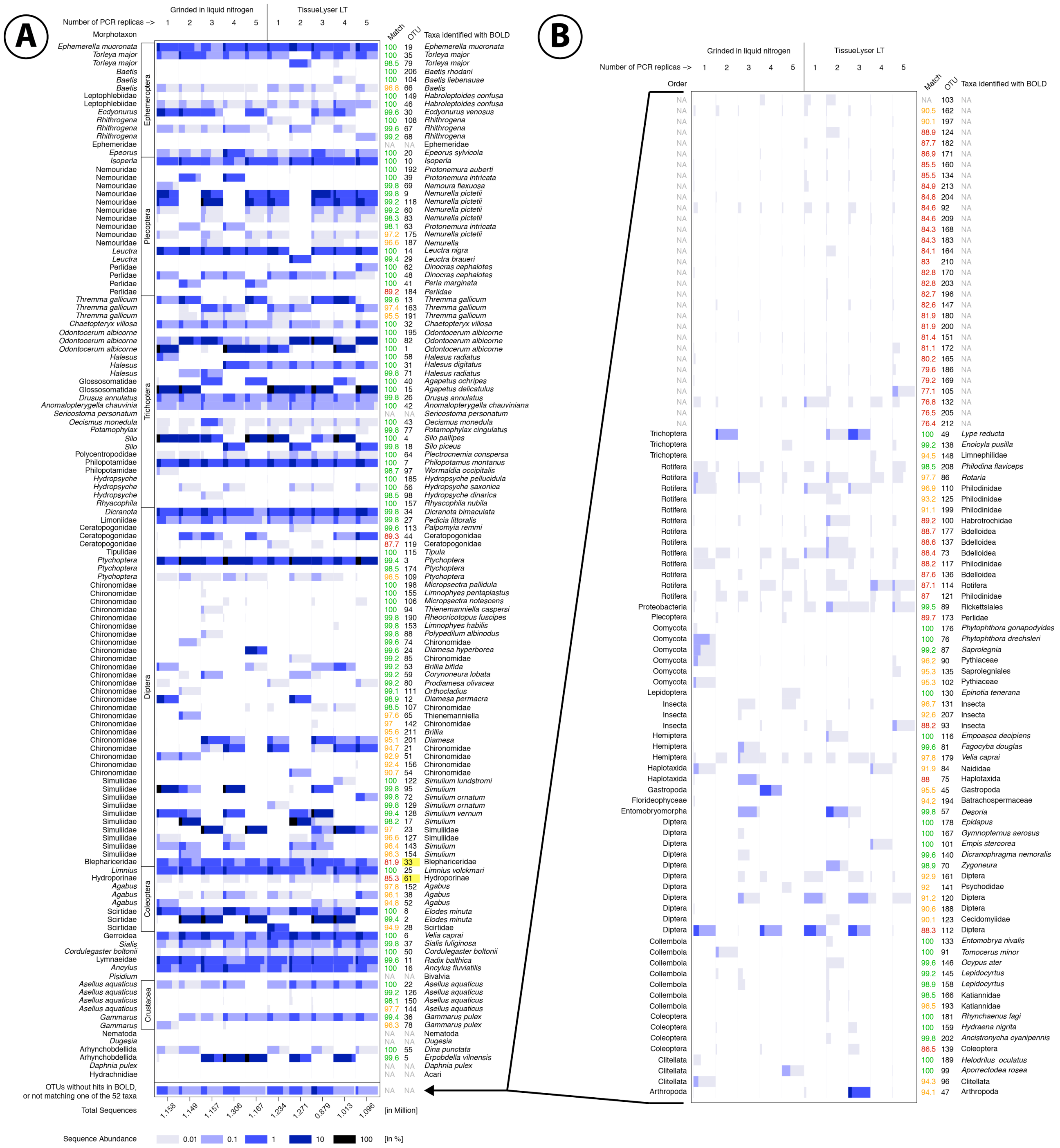

Figure S9. Experiment II: Overview of the 213 OTUs recovered across all replicates and their taxonomic assignment when compared to the BOLD database. Sequence abundance of individual OTUs can be estimated from the shading of the individual fields (see Figure 3 for a more detailed description). Sequence abundances are normalised in each of the 10 replicates. **A:** Overview of the 52 specimens included in the tier mix run, and the 120 OTUs they were assigned to. The percent identity of the respective OTU to sequences in the BOLD database is indicated as a % and matches that were verified with amino acid translations are underlaid with a yellow background behind the OTUs. **B**: Overview of the 93 OTUs that were assigned to species that did not match one of the 52 tested specimens or did not yield a match in the BOLD database.
