# Supplementary material for: Can DNA-Based Ecosystem Assessments Quantify Species Abundance? Testing Primer Bias and Biomass—Sequence Relationships with an Innovative Metabarcoding Protocol
Source: PLoS One. 2015 Jul 8;10(7):e0130324. doi: 10.1371/journal.pone.0130324 (PMC4496048; doi:10.1371/journal.pone.0130324)
Supplement: S5 Fig — Normalised sequence abundance for each stonefly. (PDF) [file pone.0130324.s005.pdf]

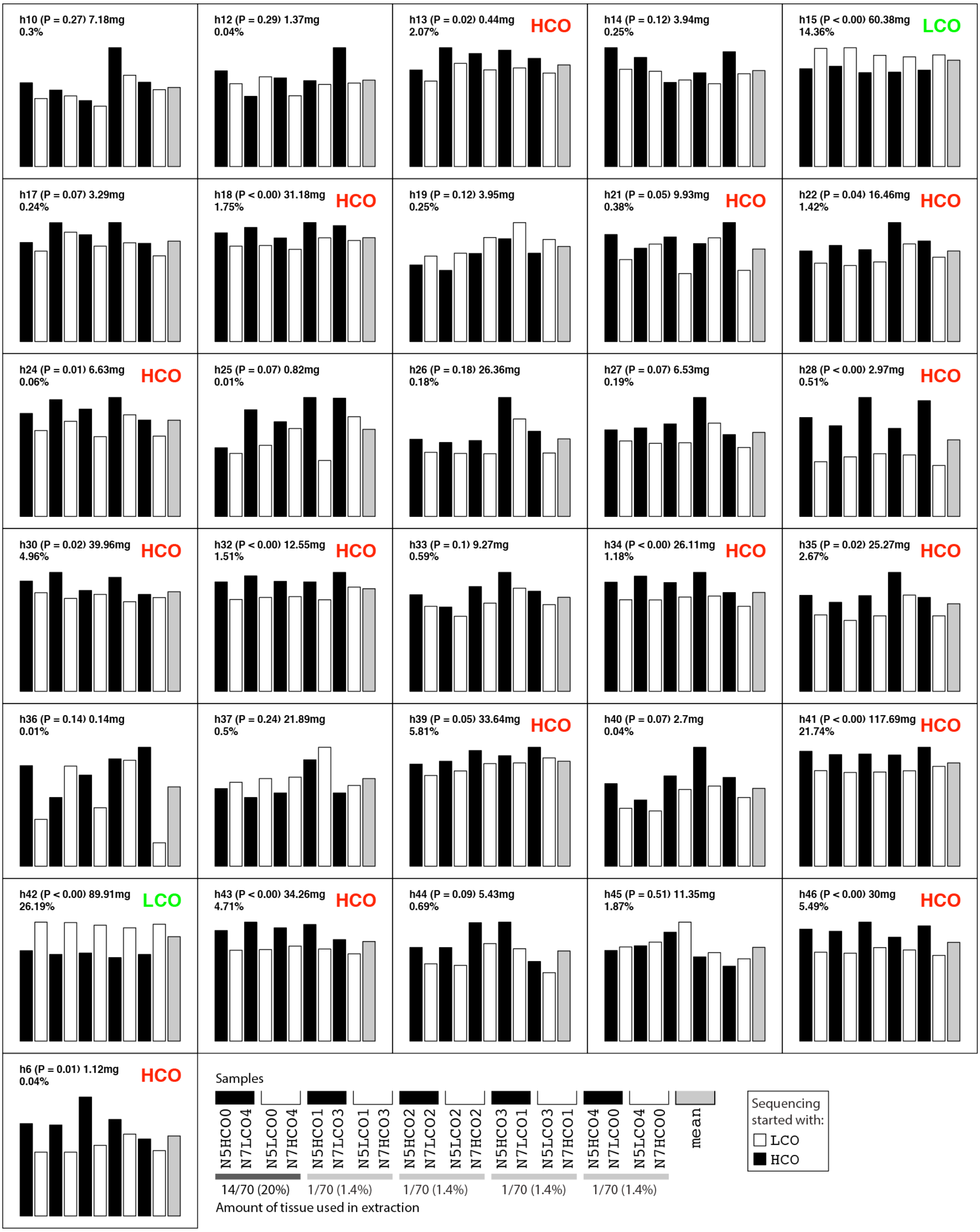

Figure S5. Experiment I: Normalised number of sequences for the 31 individual *D. cephalotes* specimens. All 10 replications per haplotype are shown as well as the average in grey in each of the 31 plots. The average (normalised) contribution of the individual specimen to the total number of sequences is shown as a % in the top left of each plot. LCO / HCO sequencing primer bias was tested with tested with a *t*-test. If a significant bias was detected, the corresponding primer name is written in the top right corner.
